# Fatty Acids Metabolism: The Bridge Between Ferroptosis and Ionizing Radiation

**DOI:** 10.3389/fcell.2021.675617

**Published:** 2021-06-24

**Authors:** Zhu-hui Yuan, Tong Liu, Hao Wang, Li-xiang Xue, Jun-jie Wang

**Affiliations:** ^1^Department of Radiation Oncology, Peking University Third Hospital, Beijing, China; ^2^Center of Basic Medical Research, Institute of Medical Innovation and Research, Peking University Third Hospital, Beijing, China; ^3^Biobank, Peking University Third Hospital, Beijing, China

**Keywords:** fatty acid metabolism, ferroptosis, irradiation, cancer, cell death

## Abstract

Exposure of tumor cells to ionizing radiation (IR) alters the microenvironment, particularly the fatty acid (FA) profile and activity. Moreover, abnormal FA metabolism, either catabolism or anabolism, is essential for synthesizing biological membranes and delivering molecular signals to induce ferroptotic cell death. The current review focuses on the bistable regulation characteristics of FA metabolism and explains how FA catabolism and anabolism pathway crosstalk harmonize different ionizing radiation-regulated ferroptosis responses, resulting in pivotal cell fate decisions. In summary, targeting key molecules involved in lipid metabolism and ferroptosis may amplify the tumor response to IR.

## Introduction

Aggressively proliferating cancer cells have an increased demand for energy and macromolecules. Thus, cancer cells prefer to expedite glycolysis utilization and glutamine consumption, as well as uptake or endogenous synthesis of lipids. Exogenous sources and endogenous lipids provide essential components to the tumor cell membrane and organelles. Fatty acids (FAs) are indispensable substrates for lipid biosynthesis and function execution, and the *de novo* synthesis of endogenous FAs is considered the major pathway for lipid recruitment by cancer cells (Menendez and Lupu, 2007). FAs are divided into saturated and unsaturated FAs according to the number of carbon-carbon bonds. Given that saturated membrane lipids are less sensitive to oxidative stress, high saturation levels of membrane phospholipids can protect cancer cells from damage induced by reactive oxygen species (ROS). Unsaturated FAs are able to subject tumor cells to oxidative stress and toxicity, and studies have recently found that metabolic stress can promote polyunsaturated FA (PUFA) binding membrane phospholipids (PLs) and make tumor cells more sensitive to ferroptosis.

Ferroptosis, a programmed cell death dependent on iron, is triggered by metabolic stress and interruption of homeostasis, especially the imbalance between the accumulation of lipid peroxide (LPO) and inactivity of antioxidant molecules, such as glutathione-dependent peroxidase (GPX4) and reduced glutathione (GSH) (Dixon et al., 2012; Yang and Stockwell, 2016). In addition, morphological, biochemical, and genetic changes in ferroptotic cells differ from those of other cells during programmed cell death. Ferroptosis cannot be induced by apoptosis inducers, even at high concentrations (Xie et al., 2016). Biochemically, the widely accepted views on the molecular mechanism of ferroptosis can be separated into three classes: cytotoxicity of PL-PUFAs, redox-active iron, and loss of lipid peroxide repair. Among these cytotoxic molecules, ROS and divalent iron ions are essential in the regulation of ferroptosis. Moreover, lipid peroxidation is considered a pivotal trigger in the final step of ferroptosis. Recently, some studies have found that ferroptosis that occurs after ionizing radiation may be a novel target for decreasing radiation resistance and promoting clinical benefits.

Ionizing radiation induces cell death by transferring energy to the molecules of the absorbing matter. Water molecules are the most important matter in the human body, and radiation can interact with water molecules to induce lethal effects in cells by generating radicals, inducing oxidative stress, or directly ionizing deoxyribonucleic acid (DNA). With the exception of target DNA, recent studies have found that ionizing radiation can change the lipid profile in several cancers, including glioma, breast, colorectal, and skin cancer (Benais-Pont et al., 2006; Bougnoux et al., 2010; Antal et al., 2014; Narayanan et al., 2015; Shaikh et al., 2017), whereas the abnormal metabolism of lipids is the central trigger for ferroptosis. Thus, increasing attention has been focused on the crosstalk between radiation, lipid metabolism, and ferroptosis.

Several studies have shown that the interplay between FA metabolism and ferroptosis is linked to oncogenesis, tumor progression, metastasis, and radiotherapy resistance. Moreover, irradiation can regulate both ferroptosis and FA metabolism. At the same time, ferroptosis may render tumor cells more vulnerable to therapies that further stress their ability to regulate redox homeostasis, thereby generating opportunities for novel therapies. Targeting substrates of lipid metabolism and regulating ferroptosis in radiotherapy could decrease toxicity and increase clinical benefits. Therefore, we reviewed the interplay between FA metabolism, ionizing radiation, and ferroptosis. We focus our discussions on the biological mechanisms by which FA metabolism might be altered by radiation, as well as the contribution it makes to radiation-regulated ferroptosis and/or the possibility of radioresistance. We also suggest directions that could guide future clinical development and research of novel combination approaches, particularly combining ferroptosis agonists with radiotherapy, as well as lipid metabolism regulators, to improve the efficacy of cancer treatment and promote radiosensitivity.

## Fatty Acid Metabolism and Ferroptosis

Metabolic reprogramming in uncontrolled-proliferation tumor cells requires lipids, proteins, and nucleotides to develop and maintain cellular structure and function, and the metabolism is significantly different from that in relative normal tissues. The considerable development of lipidomic technologies has broadened our understanding of the relevance of lipid metabolism to cancer biology.

Fatty acid metabolism has been implicated in a variety of oncogenic processes, including tumorigenesis, metastatic colonization, treatment resistance, and cell differentiation (Beloribi-Djefaflia et al., 2016). However, unlike normal cells, in which exogenous FAs play a dominant role, tumor cells have the capacity to synthesize FAs *de novo* (Ookhtens et al., 1984). The primary source of carbon for FA synthesis in cancer cells comes from glucose, which is broken down into acetyl-CoA and then citrate in the mitochondria. The *de novo* synthesis of FAs can be divided into saturated and unsaturated FAs. Unsaturated FAs bind membrane phospholipids and subject tumor cells to oxidative stress, while saturated FAs play a protective role in tumor cell biology. As most studies have revealed the relationship between unsaturated FAs and ferroptosis, the following reviews are concentrated on unsaturated FAs. Unsaturated FAs with carbon-carbon bonds can be divided into monounsaturated fatty acids (MUFAs, only one double bond) and polyunsaturated fatty acids (PUFAs, at least two double bonds).

In recent years, increasing attention has been focused on PUFA peroxides and ferroptosis. PUFAs bind to biological membrane phospholipids and tend to be oxidized to generate ferroptotic cell death under oxidative or energy stress. This form of cell death is known to be dependent on substrates that maintain redox homeostasis. Under oxidative or energetic stress, PUFA, particularly arachidonoyl (AA) and adrenic acid (AdA), is catalyzed by acyl-CoA synthetase long-chain family member 4 (ACSL4), lysophosphatidylcholine acyltransferase (LPCAT), and 15-lipoxygenase (15-LOX/ALOX15) to generate PUFA-containing phospholipids and induce ferroptosis to maintain redox homeostasis. Thus, the following section discusses the prominent effects of abnormal FA metabolism in triggering ferroptosis.

### PUFA-PL Impinges on Tumor Cell’s Susceptibility to Ferroptosis

With increasing levels of ROS, PUFA-PL is oxidized to generate PUFA-PL-OOH, which is the most important substrate for inducing ferroptosis. Among PUFA-PL, long-chain PUFAs, particularly AA or AdA, seem to be indispensable for navigating cells to ferroptosis. Indeed, genetic or pharmacological inhibition of acyl-CoA synthase 4 (ACSL4) to suppress AA or AdA esterification into PE has been shown to inhibit ferroptosis (Kagan et al., 2017). The ACSL4, LPCAT3, and ALOX family, which are involved in the synthesis of PUFA-PL-OOH, can also regulate cellular sensitivity to ferroptosis. Suppression of ferroptosis has also been observed by targeting or knockout of these enzymes (Doll et al., 2017). In breast cancer cell panels, which have diverse expression levels of ACSL4, the level of GPX4 is inversely proportional to that of ACSL4 and cell viability, and is correlated with the effect of ACSL4 in the esterification of AA and AdA into phosphatidylethanolamines (PE). Moreover, ACSL4 and ACSL3 can also catalyze AA; however, given the lower expression of free AA in the cytoplasm, other FAs can outcompete AA by combining with ACSL3. Thus, ACSL4 is a privileged enzyme that promotes ferroptosis (Doll et al., 2017). It should be noted that that regulation of ferroptosis by ACSL4 is necessary for glutathione peroxidase 4 (GPX4) inhibition, but is dispensable for the p53-mediated ferroptosis pathway, in which ALOX12 is essential for tumor suppression. The p53-mediated pathway downregulates the transcriptional level of SLC7A11 and contributes to the activation of ALOX12 (Chu et al., 2019). Following the generation of AA-CoA by ACSL4, LPCAT3 esterifies these derivatives into phosphatidylethanolamines (AA-PE and AdA-PE, also known as PL-PUFAs) in the plasma or internal membrane. PL-PUFAs are then oxidized into PL-PUFA-OOH under the assistance of ALOX15, and ultimately trigger ferroptotic cell death. Therefore, PL-PUFA-OOH acts as the main executor, triggering ferroptosis. While PUFAs stimulate cells to undergo ferroptosis, MUFA has the opposite effect.

Monounsaturated fatty acids suppresses ROS accumulation at the plasma membrane and decreases the level of PUFA-PL, inhibiting ferroptosis in an ACSL3-dependent manner (Magtanong et al., 2019). Tumor cells have the capacity to use MUFA to facilitate metastasis, and previous studies have demonstrated that some tumor types prefer to initially metastasize through the lymphatic system before metastasizing systemically through the blood. Although the mechanism underlying the phenomenon remains unclear, the Ubellacker group revealed the possible role of oleic acid, a MUFA, which can incorporate into the cell membrane as a “suit of armor” and protect cancer cells in lymph from ferroptosis and facilitate distant metastasis (Ubellacker et al., 2020). In this study, tumor cells injected intranodally were more resistant to ferroptosis due to high level of oleic acid and GSH. Furthermore, some researchers found that stearyl CoA desaturase 1 (SCD1) converts saturated FAs into MUFAs and is highly expressed in several cancers (Wang et al., 2015; Igal, 2016). In line with this, inhibition of SCD1 increases cell sensitivity to ferroptosis, with decreasing levels of CoQ10 and MUFAs (Tesfay et al., 2019).

Polyunsaturated fatty acids can be obtained from acid hydrolases from lipophage (Singh and Cuervo, 2012) and function to regulate ferroptosis. Intracellular surplus FAs do not exist as free FAs because high concentrations of free FAs are cytotoxic; thus, after executing its biological function, surplus free FAs are stored in the form of a neutral biomolecule in lipid droplets (LD), which have been shown to be abundant in tumor cells (Unger, 2002; Martin and Parton, 2006; Thiele and Spandl, 2008; Qiu et al., 2015; Klemm and Ikonen, 2020). A phase II clinical trial evaluating bortezomib monotherapy for advanced renal cancer revealed partial responses in only 12% of patients with renal cell carcinoma (Kondagunta et al., 2004). HIF-2α/PLIN2/LD-dependent lipid storage and endoplasmic reticulum stress resistance is thought to contribute to this limited response rate. In addition, LD can combine with ras-related protein rab-7a (RAB7A) and be degraded by lipophagy. In a previous study on liver cancer where HepG2 cells were treated with RSL3, the level of LD increased initially, but later decreased, and LD degradation was found to be associated with increasing levels of lipid peroxidation (Bai et al., 2019). This result highlights the balance between lipid storage and degradation, which determines the cell response to ferroptosis stress. However, the role of lipophages remains controversial. Apart from its tumor suppressor role, lipophagy-dependent degradation of lipids may provide rapidly proliferating cancer cells with energy substrates and intermediates for the synthesis of biomolecules, which contribute to the survival of tumor cells (Gomez and de Molina, 2016).

Of note, the uptake of omega-3 and omega-6 PUFAs is essential for cell function, while their precursors (linoleic acid and α-linolenic acid, respectively) are solely exogenic and cannot be provided through the *de novo* pathway (Swinnen et al., 2003). Epidemiological studies recommend that a diet rich in omega-3 PUFA is beneficial for decreasing cancer incidence. Omega-3 PUFAs can inhibit tumor cell proliferation via different pathways, including cyclooxygenase-2 (COX-2), nuclear factor-kappa B (NF-κB), Akt, and PPAR signaling pathways (Dolcet et al., 2005; Schley et al., 2005; Wu and Kral, 2005; Bai et al., 2009). In contrast, high intake of omega-6 PUFAs shows inverse effects on tumorigenesis (Pozzi et al., 2010; Panigrahy et al., 2012). Interestingly, FA transformation between pro-tumorigenesis and anti-tumorigenesis FAs dramatically promotes tumor repression. Berquin et al. (2007) used the *fat*1 transgene, which encodes omega-3 desaturase, to successfully convert most omega-6 PUFAs into omega-3 PUFAs in PTEN-knockout mice. The results suggest that pharmacological or genetic means to convert PUFAs with diverse functions may have unexpected effects on the treatment response.

In summary, considering the role of FAs, including FFA and PUFA, in ferroptosis, targeting PUFA-PL may inhibit tumorigenesis and metastasis by triggering ferroptosis.

### Disruption of Redox Homeostasis Is the Final Component in Ferroptosis Induction

Given the requirement of lipid homeostasis in normal cells, once the interruption of redox homeostasis occurs, reduced protein or molecules is initiated to scavenge excess lipid peroxidase. Glutathione (GSH) and coenzyme Q10 (CoQ10) are important antioxidants in cells.

Cysteine can act as both as a basic unit for protein translation and as an essential substrate of the antioxidative system. Two pathways have been shown to contribute to increased cysteine levels. One is the glutamate-cystine antiporter system Xc-, which can import extracellular cystine into cells and export intracellular glutamate at a 1:1 ratio, and the other is transsulfuration, which converts methionine into cysteine when extracellular sources of cysteine are limited (Zhu et al., 2019). Thus, imported cystine is reduced immediately, transformed into cysteine in cells, and used to synthesize reduced GSH under the catalysis of glutamate-cysteine ligase and glutathione synthetase. GPX4 then cleans LPO and maintains cellular redox homeostasis by using two GSH molecules as electron donors to reduce phospholipid hydroperoxides (PL-OOH) to the corresponding alcohols and leaving GSSG (oxidized GSH) as a byproduct (Yang et al., 2014). GSSG is reduced to GSH by glutathione reductase using NADPH. Thus, inhibition of the Xc- system, GSH, or GPX4 can induce intracellular accumulation of LPO and ultimately lead to cell ferroptosis.

The NAD(P)H-ferroptosis suppressor protein 1 (FSP1)-coenzyme Q10 (CoQ10, also named ubiquinone) pathway is an emerging pathway associated with ferroptosis suppression. The suppression of ferroptosis by FSP1 is mediated by CoQ10. The reduced form of CoQ10 is ubiquinol, which promotes the generation of lipid peroxyl radicals and stimulates the accumulation of lipid peroxidation using NAD(P)H. Thus, FSP1 catalyzes ubiquinol into CoQ10 and decreases the level of lipid peroxidation. FSP1-mediated suppression of ferroptosis is an independent pathway parallel to the Xc/GSH/GPX4 axis.

In short, the loss of the scavenging system for eliminating lipid hydroperoxides from PUFA-PLs is one of the hallmarks of ferroptosis.

### Peroxisomes Act as the Achilles’ Heel of FA Metabolism During Ferroptosis

**FIGURE 1:**
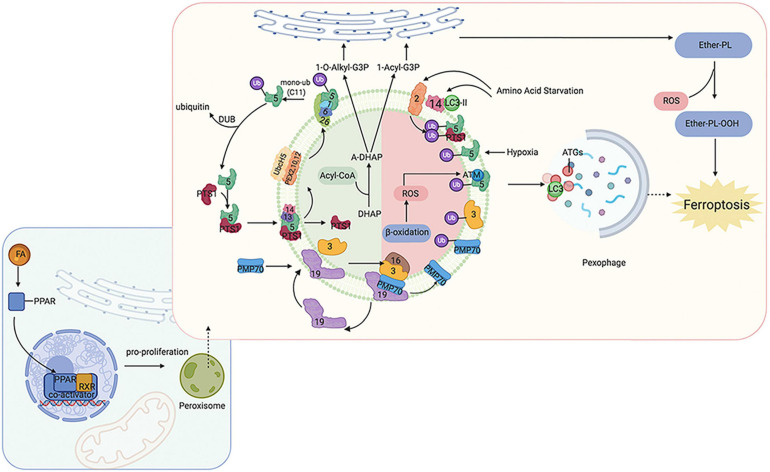
Overview of molecular and proteins, which are involved in ferroptosis, localized at the peroxisome membrane in mammals. Ferroptosis can be regulated by peroxisome synthesized ether-linked phospholipid and PPARs. G3P would be imported into peroxisomal membrane, then dehydronated by G3PDH and introduce DHAP. Then, DHAP acyltransferase (DHAPAT) uses fatty acyl CoA to acylate dihydroxyacetone phosphate (A-DHAP). Besides, fatty acyl CoA can be reduced to fatty alcohol by a peroxisomal membrane-associated fatty acyl CoA reductase with NADPH. Subsequently, acyl-DHAP is converted to alkyl-DHAP by peroxisomal fatty alcohol. Acyl- or alkyl-DHAP reductase (ADHAP) can reduce acyl-DHAP and alkyl-DHAP to 1-acyl-G3P and 1-O-alkyl-G3P, respectively. 1-acyl-G3P and 1-O-alkyl-G3P are imported into ER, and converted to diacylphospholipid or ether-linked phospholipids (ether-PL). Ether-PL can be oxidized to ether-PL-OOH, which has been considered a trigger of ferroptosis. PPARs have three receptor sub-types (PPARα, γ, and δ). PPARα and γ could facilitate MUFA synthesis to suppress ferroptosis. Although PPARδ has been reported to up-regulate the expression of ACSL3 to promote the synthesis of MUFA, PPARδ could also up-regulate ACSL4 which is a promoter of ferroptosis. PPAR, peroxisome proliferator-activated receptors; G3P, glycerol 3-phosphate; G3PDH, glycerol 3-phosphate dehydrogenase; DHAP, dihydroxyacetone phosphate; DHAPAT, DHAP acyltransferase; A-DHAP, acyl-DHAP; ADHAPR, acyl-/alkyl-DHAP reductase; PL, phospholipid; MUFA, monounsaturated fatty acids; ACSL, acyl-CoA synthetase long-chain family member; GSR, glutathione-disulfide reductase.

Peroxisomes perform many essential lipid metabolism functions, including the catabolic and anabolic processes of FAs. Peroxisomes play particularly important roles in FAO, ether-phospholipid biosynthesis, and ROS metabolism (Figure 1). Previous studies have shown that β-oxidation of FAs predominantly occurs in mitochondria;, although peroxisomes also participate in FA oxidation (Chen X. et al., 2020; Luppi et al., 2020). Although the integral compounds and full-scale function of peroxisomes remain unclear, it has been demonstrated that peroxin (PEX) families are essential components that maintain their structure and function, and peroxisome proliferator-activated receptors (PPARs), a set of three receptor subtypes (PPARα, γ, and δ) regulate a broad range of genes in many metabolically active tissues.

Peroxisomes are involved not only in catabolic processes but also in anabolic processes, such as ether phospholipids. Ether phospholipids account for 20% of phospholipids in humans, are characterized by an alkyl chain attached at the position of sn-1 (Lodhi and Semenkovich, 2014; Lodhi et al., 2015), and are the major type of ether phospholipids is plasmalogen. Given that the alkenyl-ether group of plasmalogen represents a major lipid-soluble antioxidant component, plasmalogen can scavenge ROS and mitigate cellular oxidative stress. A reduction of ether phospholipids has been observed to correlate with increasing colorectal cancer and lymph node metastasis because the high level of ROS consumes plasmalogen (Wang Y. et al., 2020). Ether was recently reported to bind phospholipids characterized by an alkyl chain attached at the sn-1 position (Lodhi and Semenkovich, 2014; Lodhi et al., 2015). Previous studies have focused on the effects of PUFA and MUFA oxidation on ferroptosis regulation; however, a recent study has found that ether phospholipids also participate in ferroptosis. Peroxisomes contribute to ferroptosis by synthesizing polyunsaturated ether phospholipids (PUFA-ePL). The downregulation of PUFA-ePL is associated with tumor cell resistance to ferroptosis peroxisomal biogenesis factor 3 (PEX3), peroxisomal biogenesis factor 10 (PEX10), peroxisomal biogenesis genes, and alkylglycerone phosphate synthase (AGPS), and fatty acyl-CoA reductase 1 (FAR1) encoding peroxisomal enzymes is considered to contribute to these effects. Alteration of PEX3, PEX10, AGPS, and FAR1 showed no connection with ACSL4 and LPCAT3 (Zou et al., 2020). In our previous work, PEX5 was found to be involved in radioresistance in hepatocellular carcinoma (HCC) (Wen et al., 2020). However, the functions of peroxisomes in regulating ferroptosis sensitivity are dispensable given that the alkenyl-ether group is not critical to ferroptosis sensitivity. PUFA-PL can complement the depletion of PUFA-ePL, and, more importantly, the ether lipid precursor 1-O-alkyl-glycerol-3-phosphate (AGP) is required for dispatch to the endoplasmic reticulum to synthesize plasmalogens (critical ethers for PUFA-ePL); however, it remains unknown how the ultimate step of PUFA-ePL synthesis is executed and regulated.

As mentioned above, PPARs play an indispensable role in peroxisome function. The expression of many genes involved in peroxisomal FA β-oxidation and proliferation is controlled by transcription factors of the PPAR family. Intriguingly, researchers have found that PPARα activity is regulated by MDM2/MDMX and drove the resistance of glioblastoma cells to ferroptosis. In addition, PPARα is associated with increased levels of saturated FAs and MUFAs (Strand et al., 2019), which may partially explain the reason for ferroptosis resistance. PPARγ also showed inhibitory effects on ferroptosis (Venkatesh et al., 2020). These findings are consistent with other publications that have shown that PPARγ can regulate SCD1 (Shi et al., 2013). SCD1 is a rate-limiting enzyme in MUFA biosynthesis; thus, PPARγ can facilitate MUFA synthesis and inhibit ferroptosis. Moreover, a recent study found inhibition of ferroptosis in tumor cells with high density due to up-regulation of E-cadherin and epithelial mesenchymal transition (EMT) (Wu et al., 2019). Pharmacological or genetic approaches to inhibit E-cadherin could rescue ferroptosis sensitivity. Interestingly, previous studies have suggested that PPARγ may increase EMT by upregulating E-cadherin (Yang et al., 2019; Kim et al., 2020). PPARδ is another isoform of PPARs, and evidence has shown that activation of PPARδ could increase ACSL3 mRNA and protein *in vitro* and *in vivo* (Cao et al., 2010). As mentioned above, ACSL3 is a negative enzyme for ferroptosis as it functions to synthesize MUFA. Moreover, in a hamster model fed with different fat diets, PPARδ was found to upregulate ACSL4 expression in liver tissue, one of the key enzymes for synthesizing PUFA-PL-OOH to trigger ferroptosis (Kan et al., 2015). These contradictory findings suggest that the response of cells to ferroptosis may be affected by different PPAR isoforms with different effects on the lipid profile. Thus, PPARs not only act as organelles to maintain redox homeostasis, but also function to regulate ferroptosis and lipid metabolism, which directly affect tumorigenesis, metastasis, and therapeutic response; thus, the specific effects under different conditions should be considered.

## Ionizing Radiation: A Versatile Regulator in the Initiation of Ferroptosis

**FIGURE 2:**
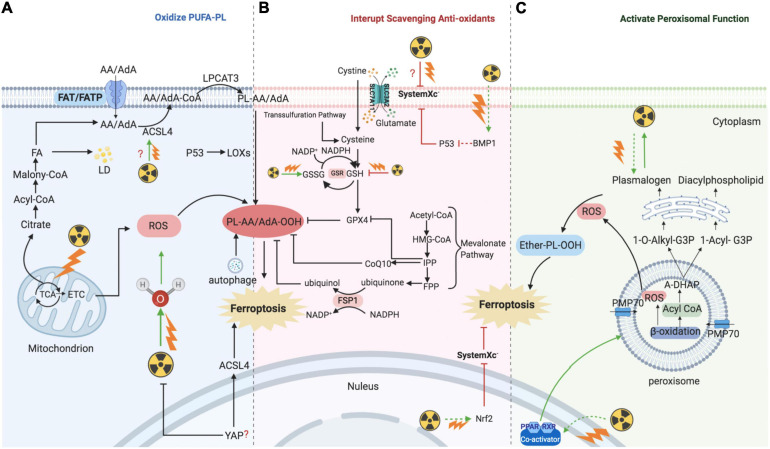
Mechanism of ionizing radiation – regulated ferroptosis. IR introducing excessive lipid peroxidation and decreasing antioxidants levels are key mechanism for regulating ferroptosis. **(A)** IR induces ferroptosis by introducing excessive lipid peroxidases. IR could up-regulate the level of ACSL4, and then ACSL4 catalyzes AA/AdA to generate Acyl-CoA. After AA generating AA-CoA by ACSL4, LPCAT3 esterifies these derivatives into AA-PL or AdA-PL in plasma membrane or internal membrane. Then, PL-AA/AdA is oxidized into PL-PUFA-OOH under the assistance of LOXs, and ultimately triggers ferroptotic cell death. In addition, IR could also provide amounts of ROS to directly oxidize PL-PUFA via ionizing water molecular or mitochondria. **(B)** IR induces ferroptosis by interrupting redox homeostasis. IR suppresses the expression of SLC7A11 and inhibits the import of extracellular cystine into cell to synthesis GSH, whereas the level of GSSG is increased. **(C)** IR induces ferroptosis by activating functions of peroxisome. IR stimulates PPARs which promote the proliferation and fatty acid β-oxidation of peroxisome. Then, peroxisome would synthesize A-DHAP which is transported to endoplasmic reticulum to synthesize plasmalogen. Plasmalogen binds PL, and under the stress of ROS, ether-PL is oxidized to ether-PL-OOH. Once the redox homeostasis is broke, ether-PL-OOH would become an inducer of ferroptotic cell death. IR, ionizing radiation; ACSL, acyl-CoA synthetase long-chain family member; AA, arachidonoyl; AdA, adrenic acid; LPCAT3, lyso-phosphatidylcholine acyltransferase; PL, phospholipid; PUFA, polyunsaturated fatty acids; LOX, lipoxygenase; ROS, reactive oxygen species; GSH, glutathione; GSSG, oxidized GSH; GPX4, glutathione-dependent peroxidase; PPAR, peroxisome proliferator-activated receptors; A-DHAP, acyl-dihydroxyacetone phosphate; FAT; FATP; FA, fatty acid; TCA, tricarboxylic acid cycle; ETC, electron transport chain; LD, lipid droplet; IPP; FPP; CoQ10, ubiquinone; FSP1, NAD(P)H-ferroptosis suppressor protein 1; PMP, peroxisomal membrane proteins.

Radiotherapy has attracted significant attention for the treatment of over 50% of all cancers, either alone or in combination with other anti-cancer therapies. Despite the expansive research on delivery techniques and dosing schedules, the outcome of radiotherapy for many cancers remains unsatisfactory, particularly with respect to radio-resistance, regardless of the original and/or acquired resistance. Thus, a deep understanding of the interaction between radiation and aberrant tumoral biological context is urgently required. In recent years, FAO-generated ferroptosis has gained increasing attention, and the understanding of the correlation between IR and ferroptosis, as well as their crosstalk in the tumor microenvironment, is increasing. According to previous studies, IR can influence several processes of FA metabolism, including synthesis (Catalina-Rodriguez et al., 2012; Martius et al., 2014; Kan et al., 2015; Koritzinsky, 2015; Benedetti et al., 2017; Gonnissen et al., 2017; Tan et al., 2018; Chuang et al., 2019; Gottgens et al., 2019; Kaur et al., 2019; Chen J. et al., 2020), transport (Zammit et al., 1989; Martius et al., 2015), oxidation, and reduction (Meister and Anderson, 1983; Martius et al., 2015; Ma et al., 2018; Ye et al., 2020), and is involved in generating FA metabolites (Richards et al., 2009; Zhong and Yin, 2015; Table 1). In other words, by taking advantage of ionizing radiation affecting lipid peroxidation, RT can further sensitize tumor cells to ferroptosis, which could be considered as a new treatment strategy to achieve better outcomes (shown in Figure 2).

**TABLE 1:** Proteins or enzymes influenced by ionizing radiation in fatty acids metabolism and ferroptosis.

	Proteins/enzymes	Expression/Level	Radio-resistance	Functions	References
**FAs transport**					
	FAT/CD36	↑	NA	Transport exogenous FAS across plasma membrane	Martius et al., 2014, 2015; Gonnissen et al., 2017
**FAs biogenesis**					
	CTP	↑	↑	Carries citrate from the mitochondria to the cytosol, associates with cancer aggressiveness	Catalina-Rodriguez et al., 2012
	ACLY	↑	↑	Key enzyme of *de novo* fatty acid synthesis, cleaves cytosolic citrate into acetyl- CoA and oxaloacetate.	Gottgens et al., 2019
	ACC	↑	↑	Converts acetyl-CoA into malonyl- CoA	Koritzinsky, 2015; Gonnissen et al., 2017
	FASN	↑	↑	Catalyzes the final step of FAs *de novo* synthesis	Chuang et al., 2019; Chen J. et al., 2020
	ACSL4	↑	↓	Catalyzes AA/AdA to generate acyl-CoA	Ye et al., 2020
**Ether synthesis**					
	PEX5	↑	↑	Receptor for PTS1, target of phosphorylation and ubiquitination,	Wang Y. et al., 2020
	PPARα/γ	↑	↑	Acts as lipid sensors to regulate peroxisomal fatty acid β-oxidation and proliferation	Benedetti et al., 2017; Kaur et al., 2019
	PPARδ	↓	NA	Acts as lipid sensors to regulate peroxisomal fatty acid β-oxidation and proliferation	Kan et al., 2015
**Fatty acid oxidation**					
	CTP1	↓	↑	The rate-limiting enzyme of FAO	Zammit et al., 1989; Tan et al., 2018
**Redox**					
	SLC7A11	↓	↓	Transport cystine into cytoplasm	Richards et al., 2009
	GPX4	↓	↓	Major antioxidant enzyme, reduces phospholipid hydroperoxides, inhibits lipid peroxidation	Ye et al., 2020
	GSH	↓	↓	The major cellular thiol participating in cellular redox reactions and thioether formation	Meister and Anderson, 1983; Ye et al., 2020
	GSSG	↓	↓		Meister and Anderson, 1983; Ye et al., 2020
**Derivation**					
	4-HNE	↑	NA	Unfold protein response (upr)	Poli et al., 2008; Zhong and Yin, 2015
	MDA	↑	NA	Modifies and cross-links proteins	Richards et al., 2009

*FA, fatty acid; IR, ionizing radiation; CTP, mitochondrial citrate transporter protein; ACLY, ATP citrate lyase; ACC, Acetyl-CoA Carboxylase; FASN, fatty acid synthase; ACSL4, acyl-CoA synthetase long-chain family member 4; PEX, peroxisomal biogenesis factor; PPAR, peroxisome proliferator-activated receptors; FAT/CD36, fatty acid transposase; CTP1, carnitine palmitoyl transferase 1; GPX4, Phospholipid hydroperoxide glutathione peroxidase; GSH Glutathione; GSSG, glutathione disulfide; MDA, malondialdehyde; 4HNE, 4-Hydroxy-2-non-enal.*

### IR Induces Ferroptotic Cell Death by Promoting Lipid Peroxidation

In addition to DNA, membranes are also considered critical targets of ionizing radiation. Several studies have supported the idea that membrane damage induced by radiation is a critical event and an initial step in triggering cell death. The plasma membrane lipid bilayer is exposed to radiation and radiation-induced ROS, resulting in lipid peroxidation, including PUFA peroxidation (Figure 2A).

After radiation exposure, PUFAs can be converted to various lipid peroxide derivatives, including malondialdehyde (MDA) (Richards et al., 2009). Moreover, 4-hydroxy-2-non-enal (4HNE), produced by lipid peroxidation, reacts easily with amino or thiol groups, and modifies and cross-links proteins, including oxidoreductases, transferases, and kinases (Poli et al., 2008). High 4HNE levels can trigger an unfolded protein response (UPR) through pathways involving protein kinase R (PKR)-like endoplasmic reticulum kinase (PERK). This signaling cascade activates the transcription factor 6 (ATF6) and inositol requirement 1 (IRE1), accompanied by JNK and p38 signaling, suggesting that 4HNE acts as an upstream modulator of the radiation-induced ROS response and endoplasmic reticulum stress (Lin et al., 2014).

As discussed above, PUFA-PL-OOH plays an important role in triggering ferroptosis. ACSL4 catalyzes AA/AdA to generate acyl-CoA and is known to be required for ferroptosis induction by generating the lipid target pool for peroxidation (Doll et al., 2017; Friedmann Angeli et al., 2019). Evidence has shown that IR can upregulate the level of ACSL4, and, in ACSL4-knockout cells, IR failed to induce ferroptosis with a decrease in lipid peroxidation. In turn, ACSL4-regulated ferroptosis can sensitize tumors to radiation. However, the mechanism by which IR regulates ACSL4 expression remains unknown. A previous study found that BMP4 could increase the level of ACLS4 and decrease the expression of p53 in epidermal growth factor receptor (EGFR)-mutant non-small cell lung cancer *in vivo* and *in vitro*(Bach et al., 2018). Moreover, IR can stimulate adipogenesis in the bone marrow by increasing the secretion of BMP4 (Bajaj et al., 2016). Notably, BMP4 may have diverse effects depending on the tumor type. For instance, BMP4 has been observed to be correlated with tumorigenesis and resistance to anti-cancer treatment by affecting FA metabolism in lung cancer (Bach et al., 2018). However, high levels of BMP4 have also been shown to be associated with promising survival rates in patients with glioma, as BMP4 promotes glioma cancer stem-like cell differentiation, and in turn, increases the tumor response to radiotherapy and chemotherapy (Xi et al., 2017).

Recently, *in vivo* and *in vitro* studies have provided mechanistic insights into the observation that cancer cells cultured at high density are resistant to ferroptosis due to the Merlin-Hippo signaling axis, which suppresses the proto-oncogenic transcriptional co-activator YAP to upregulate ACSL4 and the transferrin receptor (Wu et al., 2019; Yang and Chi, 2020). Nonetheless, if ferroptosis is consistently associated with radiosensitivity, YAP may have a positive effect on tumor cell response to IR. Indeed, several studies have demonstrated that YAP is frequently activated during the growth and progression of many solid tumors and may also confer resistance to radiotherapy. For example, YAP overexpression promotes medulloblastoma tumorigenesis, as well as the survival of cerebellar granule neuron precursor cells upon irradiation (Fernandez et al., 2012). Furthermore, YAP knockdown in urothelial carcinoma cells has been shown to increase the DNA damage response induced by γ-irradiation (Ciamporcero et al., 2016). Various biochemical and immunological methods have revealed that IR increases the expression of glucose-regulated protein 78kDa (GRP78) on the surface of cancer cells. GPR78 acts upstream of YAP/TAZ signaling and promotes migration and radiation-resistance in pancreatic ductal adenocarcinoma cells (Gopal et al., 2019). GRP78 is a member of the heat shock protein 70 (HSP70) family and acts as a centrally located sensor of stress, which responds and adapts to alterations in the tumor microenvironment. Thus, we hypothesize that ferroptosis increases the tumor response to radiation and may independently of the YAP signaling axis. Further work is needed to clarify how YAP and its upstream and downstream regulators confer resistance to radiotherapy.

In addition to regulating ACSL4 expression, it is clear that IR can directly target the tumor cell membrane PUFA-PL by generating an abundance of ROS. As discussed above, the formation of lipid peroxide by PUFA-PL-OOH plays a significant role in triggering ferroptosis, and the contribution of PUFA-PL to ferroptosis depends on oxidation by ROS. The biological effects of IR are executed by the radiolysis of water and the generation of ROS (^⋅^OH, H^⋅^, O_2_^⋅–^, H_2_, H_2_O_2_). Among these free radicals, ^⋅^OH radicals are recognized as the major ROS in the ferroptosis process because of their high activity and sufficient energy. O_2_^⋅–^ influences lipid peroxidation mainly through the Fenton reaction as opposed to directly abstracting hydrogen from pure PUFA due to the relatively low activity of O_2_^⋅–^ compared to ^⋅^OH radicals.

Ferroptosis requires ROS, and the most significant source of ROS is the tricarboxylic acid cycle (TCA cycle) and the electron transport chain (ETC). It is clear that mitochondria are one of the most important targets of radiotherapy through the generation of mitochondrial ROS (mROS). In cancer cells, mROS amplify the tumorigenic phenotype and accelerate the accumulation of additional mutations that lead to metastatic behavior. Moreover, lipid ROS initially colocalizes with mitochondria, suggesting that mROS is the major source of ROS during ferroptosis (Gao et al., 2019). Of note, suggestive evidence of the involvement of mitochondria in ferroptosis highlights that mitochondria are not only mandatory for initiating ferroptosis by providing ROS, but also cause morphological and functional damage to themselves (Xie et al., 2016; Yu et al., 2017; Gao et al., 2019; Wang H. et al., 2020). IR-mediated mROS is reportedly involved in the immune response, gene expression, nuclear DNA damage, genomic instability, activity of metabolic and antioxidant enzymes, and radiosensitivity (Kim et al., 2006, 2017; Saenko et al., 2013; Xu et al., 2018). Moreover, a previous study demonstrated that targeting mitochondrial antioxidants, including manganese superoxide dismutase (MnSOD), glutathione peroxidase 2 (Gpx2), and thioredoxin reductase 2 (TrxR2) by microRNA-17-3p remarkably sensitized prostate cancer cells to IR. In addition, the use of nanosensitizers targeting mitochondria to increase mROS accumulation can promote IR-induced cell death (Chen Y. et al., 2019). Taken together, these results suggest that IR targeting of mitochondria and promotion of mROS accumulation oxidizes PUFA-PL and makes tumor cells more vulnerable to ferroptosis. However, the role of mitochondria in regulating ferroptosis remains unclear. IR can damage mitochondrial DNA (mtDNA), whereas loss of mtDNA or removal of mitochondria does not affect ferroptosis (Dixon et al., 2012; Gaschler et al., 2018).

### IR Induces Ferroptosis by Interrupting the Scavenging Capacity of PUFA-PL-OOH

Under normal conditions, dysfunction of PUFA-PL-OOH scavenging capacity is another important means to regulate ferroptosis (Figure 2B). SLC7A11, GPX4, and GSH are essential substrates for the scavenging of peroxides. SLC7A11 expression is related to tumor invasion and metastasis via affecting the redox status or exporting glutamate in the tumor microenvironment. A previous study in prostate cancer showed that SLC7A11 expression is increased in the metastatic stromal area and is related to a low survival rate (Zhong et al., 2018). Moreover, SLC7A11 is essential for eliciting tumor formation and maintaining tumorigenicity by relieving oxidative stress in some oncogenic KRAS-mutant cancers, such as pancreatic ductal adenocarcinoma, colorectal adenocarcinoma, and lung adenocarcinoma (Lim et al., 2019). Moreover, recent studies have shown that KEAP1-NRF2 mutant lung cancer cells have high expression of SLC7A11 and are prone to resist radiation. Using ferroptosis inducers or agonists targeting SLC7A11 has the potential to sensitize radioresistant tumor cells. Moreover, abnormal expression of SLC7A11, regulated by IR, has been reported recently; high expression was found to correspond to poor survival in patients diagnosed with glioma, whereas SLC7A11 methylation indicated increased overall survival and disease-free survival. Interestingly, these associations only exist in patients who received radiotherapy and not those who did not (Ye et al., 2020). Moreover, a combination of IR and immune checkpoint inhibitors showed synergistic effects on SLC7A11 expression, and interferon-γ derived from immunotherapy-activated CD8 + T cells and IR-activated ATM both act as core regulators (Lang et al., 2019).

Recent studies revealed that p53 serves as an agonist of ferroptosis by repressing SLC7A11 and activating ALOX12 (Jiang et al., 2015; Chu et al., 2019). The diverse response of tissue to IR could contribute to the tissue specificity of p53 gene expression. A decline in radiosensitivity was accompanied by a reduction in p53 expression (Rogel et al., 1985; Komarova et al., 1997). Moreover, similar to the tumor suppression function of p53, BRCA1-associated protein 1 (BAP1) also acts as a tumor suppressor by targeting SLC7A11. Cancer genomic analysis revealed that SLC7A11 is a key target gene of BAP1 (Li C. et al., 2019). BAP1 decreases histone 2A ubiquitination occupancy on the SLC7A11 promoter and represses SLC7A11 expression in a deubiquitinating-dependent manner, leading to elevated lipid peroxidation and ferroptosis (Zhang et al., 2018). In patients with head and neck squamous cell cancer, overexpression of BAP1 was associated with higher failure rates after radiotherapy, possibly via deubiquitylation of H2Aub and modulation of homologous recombination, and was associated with poor outcomes (Liu et al., 2018). Although p53 and BAP1 have been suggested to be correlated with IR-induced SLC7A11 repression, IR-regulated ferroptosis did not affect the DNA damage response because both pharmacological (ferrostatin-1 treatment) and genetic (SLC7A11 or GPX4 overexpression) approaches did not affect phosphorylated H2AX foci formation and release of DNA damage response signals (Chk2 and p53 phosphorylation) (Lei et al., 2020).

Conversely, to gain resistance to ferroptosis, cancer cells show a tendency to upregulate SLC7A11 expression following IR treatment (Lei et al., 2020). Although we considered the increase in SLC7A11 as an adaptive response, IR-activated molecular factors should be taken into account. Nuclear factor E2-related factor 2 (NRF2) is considered an upstream signaling molecule that inhibits ferroptosis by regulating SLC7A11 under conditions of stress. NRF2 is also activated by radiation, which operates as an antioxidant adaptive response system, while activating transcription factor 4 (ATF4) is another SLC7A11-related transcription factors (McDonald et al., 2010; Chen et al., 2017). Similarly, IR has been shown to increase ATF4 in certain cancer cells (Kim et al., 2014). However, contrary to the pro-tumorigenic role of ferroptosis, the IR-regulated AFT4 increase is associated with radiotherapy sensitivity. Thus, whether IR-mediated ferroptosis depends on the repression of SLC7A11, and how IR regulates the level of SLC7A11 remains to be clarified.

The anti-oxidation molecules GSH and GSH/GSSG are also significantly decreased after radiation treatment (Ye et al., 2020), suggesting that high levels of GSH consumes ROS generated by radiation and greatly reduces the efficacy of IR. Indeed, numerous studies have revealed that depletion or inhibition of GSH can increase the tumor response to radiation (Dethmers and Meister, 1981; Bump et al., 1982; Estrela et al., 1995).

Based on these findings, IR is implicated in ferroptosis not only by forming PUFA-PL-OOH but also by inhibiting the production of reduced molecules. Conversely, the radiosensitivity of tumor cells is enhanced after treatment with ferroptosis inducers. Other anticancer treatments such as immunotherapy, show synergistic effects with IR on ferroptosis. Together, ferroptosis may provide an emergent research direction for promoting the clinical benefits of radiotherapy.

### IR Induces Ferroptosis via Activating Peroxisome

Despite the abovementioned functions of peroxisomes in FA metabolism and ferroptosis, several studies have shown the interplay between peroxisomes and ionizing irradiation and indicated a novel mechanism of IR-regulated ferroptosis (Figure 2C).

Peroxisome proliferator-activated receptors α has been shown to enhance the radiosensitivity of pancreatic cancer cells via the Wnt/β-catenin pathway, and the Wnt/β-catenin has been shown to increase intracellular bivalent iron and result in ferroptosis following asbestos exposure (Xue et al., 2018; Ito et al., 2020). Furthermore, knockout of PPARα could lead to the inhibition of radiation-induced apoptosis. The specific mechanism involved in PPARα-regulated radiosensitivity may be associated with time-dependent increases in NF-κB DNA-binding activity (Zhao et al., 2007). In human endothelial cells exposed to individual dietary FAs, linoleic acid stimulates NF-κB transcriptional activation (Toborek et al., 2002), and previous studies have found that NF-κB is abrogated by overexpression of GPX4 (Li L. et al., 2019; Brigelius-Flohe and Flohe, 2020). Similarly, in addition to PPARα, PPARγ agonists can also inhibit tumor growth by enhancing radiosensitivity (Kim et al., 2020). Moreover, activation of peroxisome function by IR may partially explain the increased expression of ACSL4 following radiation exposure. In line with this, a selective increase in ACSL4 was observed in the testes and livers of high-fat diet animals through the activation of PPARδ (Kan et al., 2015). Consistent with these findings, RT increases PPAR δ in normal tissue and decreases it in tumor tissue (Linard et al., 2008; Yang et al., 2011; Gao et al., 2012; Mangoni et al., 2017; Kaur et al., 2019). These findings imply that peroxisomes act as a dominant linker between FA metabolism and IR to generate ferroptosis.

As we have demonstrated, PEX genes are essential for peroxisomal structure and function. In mammalian cells, at least 12 PEX genes have been found to be involved in the assembly of the peroxisomal membrane, interacting with peroxisomal targeting sequences (PTSs), allowing proteins to be shuttled to peroxisomes, and acting as docking receptors for peroxisomal proteins. PEX 3 is critical for the assembly of the peroxisomal membrane and import of peroxisomal membrane proteins (PMPs). Moreover, based on Zou’s research, PEX3 contributes to ether-promoting ferroptosis, and plasmalogen, synthesized by peroxisomes, has been shown to promote ferroptosis by binding membrane phospholipids (Zou et al., 2020). Some researchers have explored the interaction, especially the synergistic effects, between IR and ether lipids, and found that ether lipids showed supra-additive cytotoxic effects with ionizing radiation and the could be considered a sensitizer to IR in radio-resistant tumor cells (Berkovic et al., 1997). Recently, CLR127, a clinical-grade antitumor alkyl phospholipid ether analog, was shown to increase the tumor response to IR *in vivo* and *in vitro* (Elsaid et al., 2018). However, the functions of other PEX genes in ferroptosis have not yet been revealed. For instance, our previous study revealed that PEX5, the cargo receptor of peroxisomes, functions to transport cargo to docking sites at the peroxisomal membrane and is highly expressed in HepG2 cells (Wen et al., 2020). Moreover, the upregulation of PEX5 in liver cancer cells was also found to be associated with radioresistance; however, whether PEX5 is a regulator of peroxisomal ferroptosis remains unclear.

Although direct evidence supporting IR-regulated ferroptosis by altering peroxisomes is scarce, considering the crosstalk between peroxisome-regulated FA metabolism, IR, and ferroptosis, it is conceivable that IR could also induce ferroptosis with the alteration of peroxisomes. However, the novel mechanism underlying the synergistic effect of IR and peroxisome requires further exploration. Taken together, research on peroxisome functions in regulating FA metabolism, ferroptosis, and radiobiology is increasingly, and the findings not only expand our knowledge on the mechanistic underpinnings of tumorigenesis and metastasis, but also highlight the need for therapeutic strategies using targeted agonists or inhibitors to be more context-dependent and personalized.

## Future Perspectives

As the interplay between lipid metabolism and ferroptosis is linked to the tumor biological context and efficiency of anticancer treatments, our knowledge of the molecular mechanism and regulation of the ionizing effect has greatly advanced in recent years. However, several key questions remain to be answered.

### Applying a Systematic and Precise Approach to Dissect the Effect of IR-Regulated Ferroptosis

Selecting a compatible dose and dose rate are important factors in radiation treatment of tumor cells. Varying the dose rate is known to alter the levels of lipid peroxidation in that lipid peroxidation shows an “inverse dose rate effect,” namely increasing lipid peroxidation at a constant absorbed dose rate, with decreasing radiation dose rate (Stark, 1991; Kumar et al., 2003; Klepko et al., 2014; Mao et al., 2016). Decreased lipid peroxidation at high dose rates is probably due to greater recombination effects when the number of positive and negative ion pairs is assembled. Thus, irradiation-related biological damage occurs rapidly without the combination of free radicals and PUFAs.

The “oxygen effect” is another explanation for the inverse dose-rate effect. The oxygen effect is widely accepted as a key element of radiosensitivity, and it is a well-established fact that IR damages significantly more tumor cells in normoxic compared to hypoxic conditions. This is partly due to molecular oxygen reacting with the induced DNA radicals to produce chemically irreparable peroxyl radicals. In addition, some studies further explored the effect of different dose rates on the oxygen effect and revealed that the oxygen effect was decreased at a lower dose rate (0.3). According to these findings, ROS generated by higher dose rate IR tends to bind DNA, while ROS introduced by lower dose rate IR is prone to peroxidize FAs. While further studies are required to validate these results, the characteristics of FA metabolism are now a reasonable option to select candidates who are unable or unsuitable for low-dose IR.

Although the “inverse dose rate effect” seems to suggest a low dose rate in order to obtain more obvious response LPO, low doses of low LET radiation (^137^Cs γ rays) delivered at a low dose-rate upregulates antioxidant defense (e.g., an increase in the level of glutathione together with upregulation of γ-glutamylcysteine synthetase expression) (Bravard et al., 1999; Kojima et al., 2002; de Toledo et al., 2006). In contrast, high LET radiation propagates oxidative stress in irradiated cells and their neighboring bystanders (Narayanan et al., 1997, 1999; Azzam et al., 2002).

In addition to the effects of dose and dose rate, other unanswered questions involve the extent to which variation in the level of expression and activity of DNA repair enzymes (DNA double-strand break repair, a predominant phenomenon of radiobiology) and proteins involved in downstream signaling after IR plus enzymes and proteins involved in ferroptosis, influence the outcome of RT. Given the highly ordered nature of the protein, a few structural changes introduced by single amino acid changes in individual proteins may significantly alter the activity of the complex. Indeed, there is increasing evidence that crosstalk among glucose, FA, and amino acid metabolism (Li and Zhang, 2016) affects cancer predisposition, and, by inference, may also modulate the response to radiation treatment.

### Heterogeneity Is Considered to Have a Significant Impact on the Outcome of IR-Regulated Ferroptosis

Cancer heterogeneity (intertumoral and intratumoral) remains a key hurdle in cancer medicine. Thus, we propose that tumoral heterogeneity, including special and temporal heterogeneity, affects the tumor response to IR-regulated ferroptosis. Different organs show different capacities and activities of FA metabolism, which may lead to diverse responses to IR-regulated ferroptosis. In addition, a single tumor over different timepoints may also present different responses. For example, hypoxia can trigger genetic mechanisms that may confer additional survival advantages to tumor cells. Hypoxia induces the expression of several genes, particularly genetic programs that are under the control of hypoxia inducible factor 1 (HIF-1). Importantly, HIF-1 can inhibit ferroptosis via a mechanism dependent on the GSH/GPX4 axis, and this process can provide selective pressure for the emergence of ferroptosis-resistant subclones. Similarly, hypoxia may select for cells with p53 mutations (since cells expressing wild-type p53 tend to undergo ferroptosis more readily), generating tumors with an anti- ferroptosis, highly malignant phenotype. Moreover, cells that are initially hypoxic may become more oxygenated during a fractionated course of RT. Following a fraction of RT, most radiosensitive aerobic cells in a tumor will be killed and the surviving fraction will be predominantly hypoxic. If sufficient time is allowed before the next fraction of radiation, some of the tumor cells will oxygenate through the process of reoxygenation, and, if this is efficient, the presence of hypoxic cells will not significantly affect the tumor response. However, the speed of reoxygenation varies widely, from a few hours in some tumors to several days in others. As a result, the treatment window of a combination of radiotherapy and ferroptosis inducer/inhibitor holds great promise for ensuring treatment efficiency.

### Targeting PUFA-PL May Lead to a New Epoch Against Malignant Disease

**FIGURE 3:**
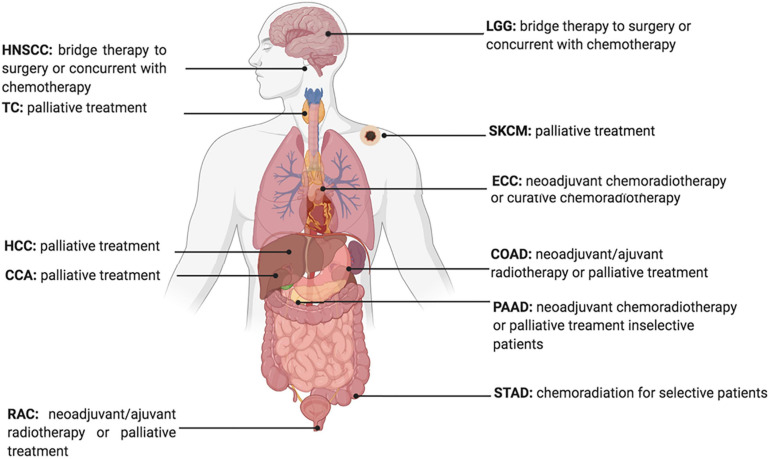
Current Status of Radiotherapy for treatment of high ACSL4 expression tumors. HNSCC, Head and Neck squamous cell carcinoma; TC, Thyroid carcinoma; HCC, hepatocellular carcinoma; CCA, cholangiocarcinoma; RAC, Rectum adenocarcinoma; ECC, Esophageal carcinoma; LGG, Brain Lower Grade Glioma; STAD, Stomach adenocarcinoma; PAAD, Pancreatic adenocarcinoma; COAD, Colon adenocarcinoma; SKCM, Skin Cutaneous Melanoma.

Developing therapeutic strategies to target lipid metabolism and ferroptosis in cancers may be of great interest for anticancer treatment. For example, erastin, a widely used ferroptosis inducer that influences the ferroptotic process by inhibiting the Xc^–^ Xc-system directly and decreasing the intake of cystine, is reported to decrease the radio-resistance of non-small cell lung cancer (Pan et al., 2019). Furthermore, given the increased expression of ACSL4 in tumor cells treated with IR, ACSL4 could be used as an effective biomarker to show tumor response to IR (Figure 3; Lei et al., 2020).

The contribution of ferroptosis inducers or inhibitors has been reported to alter the clinical efficacy of radiotherapy. Indeed, the erastin-promoting effects of radiosensitivity indicate that combining radiotherapy and ferroptosis inducers might increase tumor response (Pan et al., 2019; Shibata et al., 2019). Moreover, it has been reported that ferroptosis is associated with radiotherapy complications. The GPX4 level was decreased in radiated lungs, and liproxstatin-1 could protect lung tissue from radiation-induced lung fibrosis by activating the Nrf2 pathway and increasing GPX4 (Li X. et al., 2019). Thus, selecting candidates that are prone to radiation damage induced by ferroptosis, and combining ferroptosis inhibitors and radiotherapy to avoid injury would introduce a new therapeutic approach to enhance treatment efficacy.

Ferroptosis is also associated with several immunological processes, and immunotherapy combined with IR appears to synergistically suppress SLC7A11 and promote tumor cell ferroptosis (Lang et al., 2019). Thus, this may offer new options for further understanding and resolving issues in immunotherapy and radiotherapy.

### Outstanding Issues for Future Research

Despite the rapid growth of research on ferroptosis-based cancer therapy, some challenges remain to be overcome. First, direct evidence supporting the effects of MUFA on tumor cell proliferation is insufficient, and some evidence has shown that converting pro-tumorigenesis FAs (e.g., omega-6 PUFA) into anti-tumorigenic FAs (e.g., omega-3 PUFA) inhibits tumor growth. Second, evidence has shown that the most specific signals of ferroptosis in humans remain sparse. Numerous experiments have measured 4-HNE and MDA, two final degradation products of LPO, to determine the level of ferroptosis in human tissue. In addition, Li W. et al. (2019) found that the recruitment of neutrophils to coronary vascular endothelial cells is initiated through ferroptosis in heart transplantation. However, neutrophils are not unique markers for predicting the cellular response to ferroptosis. Abnormal recruitment of neutrophils can also be induced by inflammation, cancer, treatment approaches, or certain physical conditions. Therefore, considering future clinical applications, more accurate, specific, convenient, and economic signals are required. Third, some classic pathways, such as Wnt, P53, NF-κB, mTOR, MAPK, and BMP, all of which are involved in tumor growth, have been reported to be involved in ferroptosis. However, how these signaling pathways regulate IR-regulated ferroptosis, and how FA metabolism changes following their activation, are questions for further work, the answers to which are necessary to resolve the relationship between radiation and ferroptosis. Finally, given that mitochondria are targets of radiation, whether mitochondrial functions are complemented by peroxisomes, another organelle involved in FA metabolism following IR, needs to be explored.

## Conclusion

Emerging research has progressively increased the understanding of ferroptosis mechanisms, interpreted ionizing radiation effects on ferroptosis, and revealed the interplay among irradiation, ferroptosis, and lipid metabolism. In the future, more efforts should be focused on the crosstalk between ferroptosis and radiotherapy in terms of different metabolic states, not only from the lipid metabolic profile, but from a broader view to integrate the entire metabolic environment at a given dose and given cancer type by a series of comprehensive studies. The interactome strategy, including the novel techniques and probes in the field of chemoproteomics, will deepen our understanding of this subject.

## Author Contributions

Z-hY and TL wrote the original draft of the manuscript. Z-hY, TL, L-xX, and J-jW wrote, reviewed, and edited the manuscript. All authors have read and agreed to the published version of the manuscript.

## Conflict of Interest

The authors declare that the research was conducted in the absence of any commercial or financial relationships that could be construed as a potential conflict of interest.
